# Prediction of In-Flight Particle Properties and Mechanical Performances of HVOF-Sprayed NiCr–Cr_3_C_2_ Coatings Based on a Hierarchical Neural Network

**DOI:** 10.3390/ma16186279

**Published:** 2023-09-19

**Authors:** Longen Gui, Botong Wang, Renye Cai, Zexin Yu, Meimei Liu, Qixin Zhu, Yingchun Xie, Shaowu Liu, Andreas Killinger

**Affiliations:** 1 School of Mechanical and Electrical Engineering, Soochow University, Suzhou 215137, China; 2 Institute for Manufacturing Technologies of Ceramic Components and Composites (IMTCCC), University of Stuttgart, Allmandring 7b, 70569 Stuttgart, Germany; 3 School of Automobile and Transportation Engineering, Guangdong Polytechnic Normal University, Guangzhou 510665, China; cairenye@gpnu.edu.cn; 4 National Engineering Laboratory for Modern Materials Surface Engineering Technology, Institute of New Materials, Guangdong Academy of Science, Guangzhou 510650, China; 5 School of Mechanical Engineering, Suzhou University of Science and Technology, Suzhou 215009, China; 6 CNRS, INRAE, Centrale Lille, UMR 8207—UMET—Unité Matériaux et Transformations, Université de Lille, 59000 Lille, France; shaowuliu93@163.com

**Keywords:** high-velocity oxygen fuel spray, NiCr–Cr_3_C_2_ coatings, machine learning, physical information neural network, convolutional neural network

## Abstract

High-velocity oxygen fuel (HVOF) spraying is a promising technique for depositing protective coatings. The performances of HVOF-sprayed coatings are affected by in-flight particle properties, such as temperature and velocity, that are controlled by the spraying parameters. However, obtaining the desired coatings through experimental methods alone is challenging, owing to the complex physical and chemical processes involved in the HVOF approach. Compared with traditional experimental methods, a novel method for optimizing and predicting coating performance is presented herein; this method involves combining machine learning techniques with thermal spray technology. Herein, we firstly introduce physics-informed neural networks (PINNs) and convolutional neural networks (CNNs) to address the overfitting problem in small-sample algorithms and then apply the algorithms to HVOF processes and HVOF-sprayed coatings. We proposed the PINN and CNN hierarchical neural network to establish prediction models for the in-flight particle properties and performances of NiCr–Cr_3_C_2_ coatings (e.g., porosity, microhardness, and wear rate). Additionally, a random forest model is used to evaluate the relative importance of the effect of the spraying parameters on the properties of in-flight particles and coating performance. We find that the particle temperature and velocity as well as the coating performances (porosity, wear resistance, and microhardness) can be predicted with up to 99% accuracy and that the spraying distance and velocity of in-flight particles exert the most substantial effects on the in-flight particle properties and coating performance, respectively. This study can serve as a theoretical reference for the development of intelligent HVOF systems in the future.

## 1. Introduction

High-velocity oxygen fuel (HVOF) spraying is a high-energy thermal spraying process for depositing protective coatings with anticorrosion, anti-erosion, and anti-wear properties [1,2,3]. In the HVOF technique, a mixture of fluid/gaseous fuel and oxygen is ejected through a Laval nozzle at supersonic speeds into a combustion chamber [4]. At extremely high temperatures and pressures, the resultant gas heats and accelerates the feeding powder to form the final coating on the substrate [5]. The in-flight particle properties (temperature and velocity) critically affect the performances of HVOF-sprayed coatings [6,7] and depend on the parameters of the HVOF process, including the standoff distance, oxygen flow, and fuel flow [8]. However, owing to the complex physical and chemical phenomena involved in the HVOF technique, designing spraying parameters that optimize the coating performances is challenging. HVOF-sprayed coatings have been predicted and optimized through various approaches, such as experimental design, numerical simulation, and machine learning (ML) [9,10,11,12,13].

Recently, statistical optimization methods, including the Taguchi method and numerical simulations, have been widely employed to establish polynomial regression equations and clarify the influence of the process parameters on the coating properties. For example, Nguyen et al. [14] used the Taguchi method to investigate the optimal process parameters of HVOF-sprayed WC-16Co coatings. However, as the performance of coatings is affected by multiple sets of HVOF process parameters, these methods do not make accurate predictions of the coating performance based on experimental designs, thereby substantially limiting their applicability. Nevertheless, the entire HVOF process, including the variation patterns of flame pressure, temperature, velocity, Mach number, combustion composition, temperature, velocity, and motion trajectory of WC-12Co particles, has been analyzed using numerical models [15]. However, it should be mentioned that in metal–ceramic composites, as one of the most studied HVOF-sprayed coatings, such as NiCr–Cr_3_C_2_ and WC-Co, carbide (WC and Cr_3_C_2_) powders are susceptible to different degrees of decomposition reaction under various HVOF spraying parameters [16]. There is no doubt that the change in composition of in-flight particles will significantly affect the deposition behavior of each particles, influencing the temperature and velocity of in-flight particles. As such, the complex evolution procedures (e.g., phase transformation, dissolution, and evaporation of some elements or compositions) make it very difficult to model the real behaviors of the particles via numerical simulations. Consequently, neither the Taguchi method nor the numerical simulation-based modeling technique can accurately predict the spraying parameters and coating performance.

Machine learning (ML) techniques can overcome the abovementioned technical difficulties of the Taguchi and numerical modeling methods [17]. Artificial neural networks (ANNs) with flexible structures and powerful learning ability are most commonly proposed for the prediction and optimization of thermal spraying processes. In 2004, ANN implementation in the thermal spray process was pioneered by the LERMPS laboratory [18]. They modeled the plasma spraying technique using an ANN and then analyzed and predicted the coating characteristics, including the percentage of unmelted particles, porosity, and phase composition [18,19,20,21]. Choudhury also trained an ANN model for predicting the in-flight particle characteristics of an atmospheric plasma spraying process [22]. Moreover, he investigated the relation between these characteristics and the properties of plasma-sprayed 8YSZ electrolyte coatings [23]. Recently, researchers have explored ML algorithms and neural networks that predict the performance of TiO_2_ coatings deposited using a novel plasma spraying technique called suspension plasma spraying [24]. However, research on neural network-based predictions of the HVOF spraying process and HVOF-sprayed coatings is limited compared to that on neural network-based predictions of plasma spraying [13,25].

As mentioned earlier, researchers have predicted the particle or coating properties in case of the plasma spraying process, but studies on the relationships among the HVOF process parameters, intermediate variables (i.e., temperature and velocity of the in-flight particles), and coating properties are limited. Moreover, most prediction models for both HVOF and plasma spraying in-flight particle characteristics are based on ANNs [13,18,19,20,21], which tend to be overfitted when the input data are limited.

Convolutional neural networks (CNNs) can automatically mine the potential pattern features, which is potentially less time consuming than accumulating experience and professional knowledge [17]. Unlike ANNs, CNNs employ a weight-sharing feature that reduces the number of trainable network parameters, enhancing the generalization of the model and avoiding or weakening overfitting [26]. Moreover, a CNN model is a powerful tool for pattern recognition in various computational materials problems characterized by local interactions [27]. Using a CNN-based approach, Zhang et al. [28] attempted to improve predictions of the material removal rate in chemical mechanical polishing. Lu et al. [29] developed a CNN model that rapidly and accurately predicts the amorphous formation ability of various amorphous alloys; they reported a prediction accuracy of 0.71693, more than 19% higher than those of 13 standards. CNN-based studies are currently few and limited to air-plasma spraying and air plasma-sprayed coatings. The relation between in-flight particles and the spraying parameters has been implicitly established and analyzed in a previous study [17]. Thus, developing CNN models of the HVOF process and HVOF-sprayed coatings is desired and should be attempted.

However, the ANN and CNN models are based on blackbox algorithms which obscure their interpretability [30], i.e., ANNs and CNNs generate their prediction and relation results without considering the basic physical models of the HVOF process. The physics-informed neural network (PINN) is a hybrid physical–statistical ML method that embeds automatic differentiation and partial differential equations into the loss function of neural networks [31]. Moreover, the PINN combines physical models with neural network regression to compensate for the poor transferability of traditional ML techniques, such as ANN and CNN, to unknown structures. In thermal fluid studies, the PINN has been used to solve momentum and energy transport governing equations [32] and to accurately predict the velocity and temperature of particles [33,34]. In addition to thermal fluid problems, PINNs have been applied to various material applications. Zhang [35] found that among various deep neural networks and traditional ML models, the PINN most accurately predicts the creep–fatigue life of 316L stainless steel. To the best of our knowledge, a PINN model has never been applied to thermal spraying, which is characterized by complex flow fields and thermal fields during deposition.

**Figure 1:**
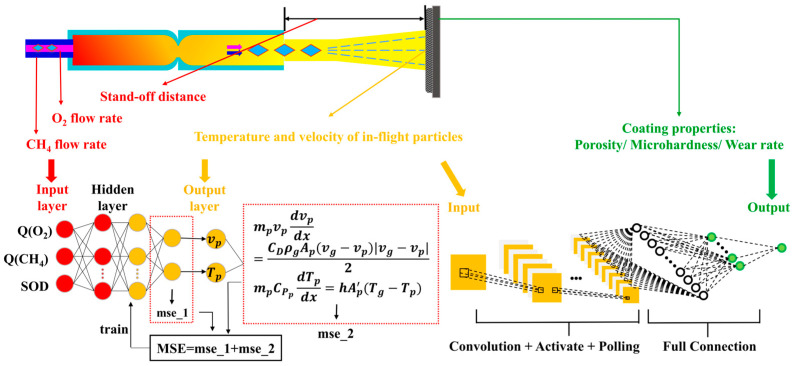
The framework of this work.

This study attempts to optimize HVOF-sprayed NiCr–Cr_3_C_2_ coatings and predict their in-flight particle properties based on the HVOF process parameters and the properties of the NiCr–Cr_3_C_2_ coatings. The HVOF process and HVOF-sprayed coatings are analyzed using a PINN–CNN hierarchical neural network that synergistically combines the advantages of the PINN and CNN. The primary objective is to propose a layered learning model that utilizes the PINN to construct the first layer for predicting the HVOF in-flight particle properties, which is followed by that of a second layer using a CNN for prediction of coating properties. The framework is illustrated in Figure 1. The relative importance of the spraying parameters with respect to the properties of in-flight particles and coating performance are then evaluated using a random forest (RF) model. Results indicated that spraying distance and in-flight particle velocity most considerably affect the in-flight particle properties and coating performance, respectively. Furthermore, compared with experimental data, the temperature and velocity of in-flight particles as well as the coating properties (cross-sectional porosity, wear resistance, and microhardness) are predicted with up to 99% accuracy.

## 2. Experimental Procedures

### 2.1. HVOF Spray Process Parameters

The raw material used herein is a commercially available NiCr–Cr_3_C_2_ powder (METCO 81 VF-NS: Oerlikon Metco AG, Wohlen, Switzerland) with particle size ranging from 5 μm to 45 μm. HVOF spraying experiments were conducted using an in-house fabricated Diamond Jet spraying system. The gun was guided by a si*x*-axis industrial robot (IRB2600-20: ABB, VÄSTERÅS, Sweden); a sandblasted 316L stainless steel plate (Ø 25 mm × 10 mm) was used as the substrate. During the experiments, three process parameters, namely the oxygen flow rate (Q(O_2_)), fuel flow rate (Q(CH_4_)), and standoff distance (SOD), were varied. In addition, in-flight particles were characterized during HVOF spraying using a commercial diagnostic system, AccuraSpray G3 (Tecnar, St-Bruno, PQ, Canada). The specific spraying process parameters are listed in Table 1.

### 2.2. Coating Microstructure Characterization

The porosity of the coating was determined through analyzing its cross-sectional microstructure via optical microscope (Nikon, Japan). Over 10 consecutive images were captured, and the average value was calculated using ImageJ imaging software (version: 1.8.0_112). The microhardness of the coatings was evaluated using a Vickers microhardness tester (Leiz—Wetzlar, Germany) with a load of 300 gf and a dwell time of 25 s on a cross-section of the coating. Twenty random indentations were made, and an average microhardness value was obtained for each coating. Wear rates were determined through performing dry sliding wear tests using a CSEM tribometer (Neuchatel, Switzerland) at an atmospheric temperature of 15–20 °C and a humidity of 40–50%. The paired material used comprised Al_2_O_3_ balls (diameter = 6 mm) with a load of 5 N, a rotation radius of 7 mm, a linear rotation speed of 10 mm/s, and a sliding distance of 500 m. The cross-sectional profile of the wear track was measured using a profilometer (Altisurf 500, Thonon-les-Bains, France), and 10 contour measurements were performed to obtain the average wear rate. The wear rate is denoted by K as expressed in Equation (1).

(1) $$K=\frac{\Delta V}{SF_{N}}$$

Here, ΔV denotes the volume loss of the material, S denotes the sliding distance, and $F_{N}$ denotes the applied normal load.

For the above characterization and performance tests, all specimens were prepared via grinding using P220 SiC paper (Struers, Champigny-sur-Marne, France) and MD-Largo disks (Struers, Champigny-sur-Marne, France), followed by polishing with a 3-μm diamond suspension and a 0.04-μm nondrying colloidal silica suspension.

## 3. Hierarchical PINN-CNN Models and Their Implementations

### 3.1. Feature Selection Based on the Random Forest (RF) Model

**Figure 2:**
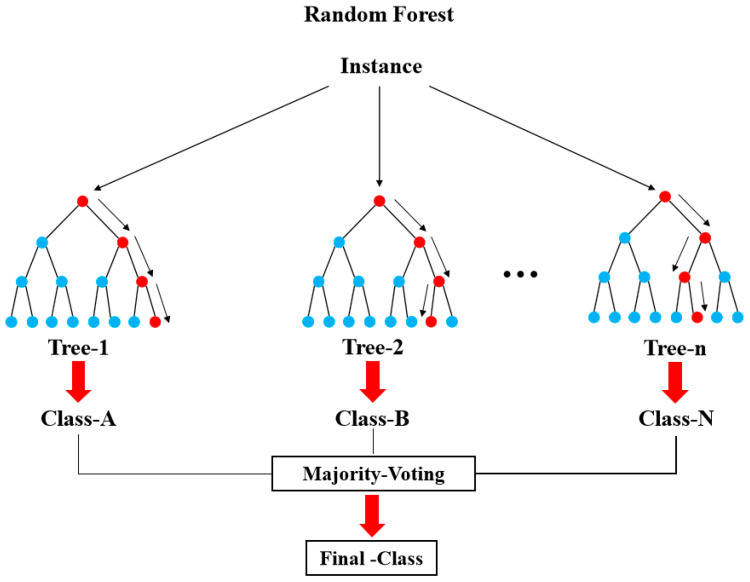
The random forest model structure.

The HVOF spraying process involves numerous feature variables. To avoid compromising prediction accuracy, we applied the RF feature selection algorithm to identify essential features for prediction. The final classification outcome was determined through a voting process among the base classifiers. Integrating multiple decision trees in the RF model renders it more stable than a standalone decision tree model, particularly in classifying complex feature variables and unbalanced categories. This property results in more accurate predictions and reduces the likelihood of overfitting (Figure 2).

In the RF model, noisy but important features can substantially affect classification accuracy. Therefore, we used feature importance as a criterion for selecting the features of the RF algorithm. The feature importance metric $\bar{D_{j}}$ for feature $X_{j}$ is given by Equation (2):

(2) $$\bar{D_{j}}=\frac{1}{B}\overset{B}{\underset{t=1}{\sum }}(R_{b}^{oob}-R_{bj}^{oob})$$

where *B* denotes the number of training samples, $R_{b}^{oob}$ denotes the number of correct classifications counted by the decision tree for the out-of-bag data, and $R_{bj}^{oob}$ denotes the number of correct classifications counted when the decision tree classifies the out-of-bag data after perturbation.

### 3.2. Data Collection and Preprocessing

**Figure 3:**
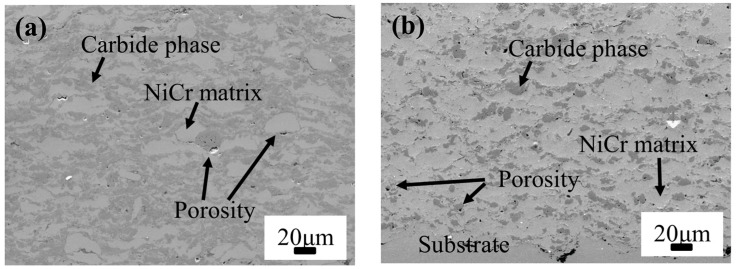
Typical lamellar cross-section microstructures of sample (**a**) No. 5 and (**b**) No. 35.

The dataset utilized in the experiments comprise 320 data points from 40 sets of spraying experiments and corresponding characterizations and performance tests on HVOF-sprayed NiCr–Cr_3_C_2_ coatings. The experiments include monitoring in-flight particles (temperature and velocity) and coating characterization tests (e.g., porosity, wear resistance, and microhardness). The spraying parameters, characteristics of the in-flight particles, and coating performances are summarized in Table 2. Additionally, all these HVOF-sprayed NiCr–Cr_3_C_2_ coatings exhibited typical lamellar structure. Taking the No. 5 and No. 35 samples as examples, different NiCr/Cr_3_C_2_ ratio and lamellar structures with porosity were observed in Figure 3, where the NiCr matrix is light gray and Cr_3_C_2_ is in dark gray. The PINN considers the standoff distance (SOD), oxygen flow rate (Q(O_2_)), and fuel flow rate (Q(CH_4_)) as inputs and particle in-flight velocity and temperature as outputs. The PINN outperforms current ML approaches used in thermal spraying through incorporating physical information regarding the system. In addition, it reduces the dataset required to uncover complex prediction–response relations, shortens training time, and rationalizes the mechanism of the spraying process. Therefore, we used the PINN as the first layer of the layered neural network model to predict particle in-flight velocity and temperature through inputting standoff distance (SOD), oxygen flow rate (Q(O2)), and fuel flow rate (Q(CH4)).

In the second layer of the neural network model, we used a CNN with in-flight particle velocity (V) and temperature (T) as inputs and microhardness, porosity, and wear rate of coatings as final outputs. Before training the model, we normalized it according to Equation (3) through setting the variable input range between one and two to prevent computational errors resulting from different parameter magnitudes. To prevent overfitting, we randomly divided the data into the training and validation sets, with proportions of 80% and 20%, respectively.

(3) $$X_{NORM}=\frac{X-X_{MIN}}{X_{MAX}-X_{MIN}}$$

**Figure 4:**
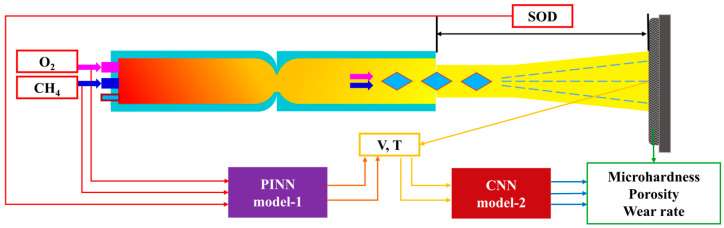
Specific representation of data in the model.

Here, $X_{NORM}$ denotes the normalized value, X denotes the experimental value, $X_{MAX}$ denotes the maximum experimental value, and $X_{MIN}$ denotes the minimum experimental value. Specific representation of the data in the model is shown in Figure 4.

### 3.3. PINN Model: First-Layer Building, Training, and Validation

The layered machine model was implemented in PyTorch, a deep learning framework based on Torch, and developed using Python. The first layer of the model employs a PINN with transfer functions, including functions from the input layer to the first hidden layer, between hidden layers, and from the second hidden layer to the output layer set to *tansig*, *logsig*, and *purelin*, respectively. In addition, proportional conjugate gradient backpropagation (*trainscg*) was selected as the training function.

**Figure 5:**
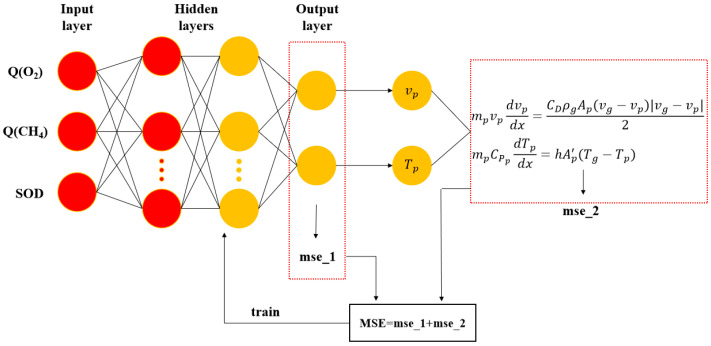
PINN model architecture diagram.

As there were three input variables and two target variables in the dataset, the number of neurons in the input layer was set to three and the number of neurons in the output layer was set to two. However, no general rule exists for determining the exact number of neurons in the hidden layer. Typically, the fewer the hidden layers and number of neurons in the hidden layers, the more lightweight the model. Therefore, in this study, a neural network model with two hidden layers was selected and the number of neurons in each hidden layer was set to 10 after simulation and comparison based on the prediction results. Figure 5 depicts the PINN model architecture diagram.

Herein, the neural network model is trained using the backpropagation method, a commonly used algorithm for learning multilayer networks. The training is supervised, and the inputs are forwarded through a transfer function to calculate the error between the model output and actual experimental data. This error, denoted as *mse*_1, is optimized during training to improve network performance. Additionally, physical formulas are embedded, such as those for particle velocity and temperature, using Lagrangian methods. The loss functions for $v_{p}$ and $T_{p}$ in Equations (4) and (5) are associated with an error of *mse*_2. To minimize the errors, *mse*_1 and *mse*_2 are backpropagated to adjust the network weights and bias values. Two metrics were used to evaluate the CNN results in this study: the mean absolute error (MAE) of Equation (6) and the decidability factor ($R^{2}$) of Equation (7). Smaller MAE values and a larger $R^{2}$ indicate a better model fit and higher prediction accuracy, respectively.

(4) $$m_{p}v_{p}\frac{dv_{p}}{dx}=\frac{C_{D}\rho_{g}A_{p}\left(v_{g}-v_{p}\right)\left|v_{g}-v_{p}\right|}{2}$$

(5) $$m_{p}C_{P_{p}}\frac{dT_{p}}{dt}=hA_{p}^{\prime }\left(T_{g}-T_{p}\right)$$

In Equation (4), $v_{p}$ denotes the velocity of the particle flight, $m_{p}$ denotes the mass of the particle, $v_{g}$ denotes the gas velocity, $C_{D}$ denotes the drag coefficient, $A_{p}$ denotes the particle cross-sectional area, $\rho_{g}$ denotes the gas density, and x denotes the axial position. In Equation (5), $T_{p}$ denotes the particle temperature, $A_{p}^{\prime }$ denotes the particle surface area, $C_{P_{p}}$ denotes the particle heat capacity, $T_{g}$ denotes the gas temperature, and h denotes the heat transfer coefficient.

(6) $$MAE=\frac{1}{n}\overset{n}{\underset{i=1}{\sum }}\frac{\left|y_{i}-\hat{y_{i}}\right|}{y_{i}}$$

Here, $y_{i}$ denotes the true value, $\hat{y_{i}}$ denotes the predicted value (*i* = 1, 2, …, *n*), and *n* denotes the number of samples.

(7) $$R^{2}=1-\frac{\sum_{i=1}^{N}{\left(t_{i}-a_{i}\right)}^{2}}{\sum_{i=1}^{N}{\left(t_{i}-{\overset{=}{t}}_{i}\right)}^{2}}$$

Here, $t_{i}$ denotes the experimental results, $a_{i}$ denotes the predicted result, ${\overset{=}{t}}_{i}$ denotes the mean of the experimental results, and N denotes the number of datasets.

### 3.4. CNN Model: Building, Training, and Validation of the Second Layer

**Figure 6:**
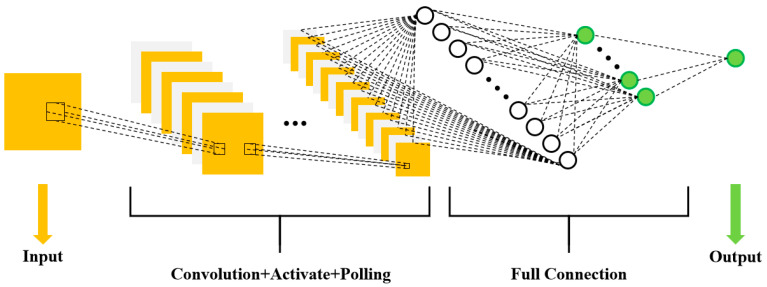
The basic architecture of a CNN.

In this work, we use a CNN to construct the second layer of our model. We evaluate and compare the predictive performance of the CNN model using the same evaluation metrics as the PINN. The CNN mainly comprises a convolutional layer, pooling layer, and fully connected layer (Figure 6).

Herein, we propose a novel architecture wherein the input layer is the predicted particle flight speed and temperature obtained from the PINN model. The convolutional layer performs layer-by-layer feature extraction of the input signal, and the obtained features are then mapped to the output layer using the fully connected layer. The output layer predicts the microhardness, porosity, and wear rate of the coating. The convolutional layer is the central component of the CNN and calculates feature values from the input data. The specific calculation is expressed in Equation (8).

(8) $$c\left(t\right)=f*\omega =\underset{p=1}{\sum }f\left(p\right)\times \omega \left(p-q\right),0\leq m<M,0\leq n<N$$

Here, f denotes the input signal guide, ω denotes the convolution kernel, n denotes the total number of signals, and q denotes the size of the convolution kernel.

The primary function of the pooling layer is to decrease the dimensionality of the data, reduce computation, and prevent overfitting while enhancing the robustness of the system. Because the dataset is small, the network comprises only two convolutional layers and lacks a pooling layer.

The fully connected layer is a shallow perceptron that establishes complete connections between the output layer and the data acquired after multiple convolution and pooling operations. Finally, the *Softmax* activation function classifies the results obtained in the output layer, computed as shown in Equation (9):

(9) $$f\left(x^{\left(i\right)}|\theta \right)=\left[\begin{array}{c} p\left(y^{\left(i\right)}=1|x^{\left(i\right)},\theta \right)\\ \vdots \\ p\left(y^{\left(i\right)}=k|x^{\left(i\right)},\theta \right) \end{array}\right]=\frac{1}{\sum_{j=1}^{k}e^{\theta_{j}^{T}x^{\left(i\right)}}}\left[\begin{array}{c} e^{\theta_{1}^{T}x^{\left(i\right)}}\\ \vdots \\ e^{\theta_{k}^{T}x^{\left(i\right)}} \end{array}\right]$$

where k denotes a particular classification, $\theta_{1}^{T}x^{\left(i\right)}$ denotes the value of that classification, $p\left(y^{\left(i\right)}=k|x^{\left(i\right)},\theta \right)$ denotes the probability of the kth category, and θ denotes the weight.

**Figure 7:**
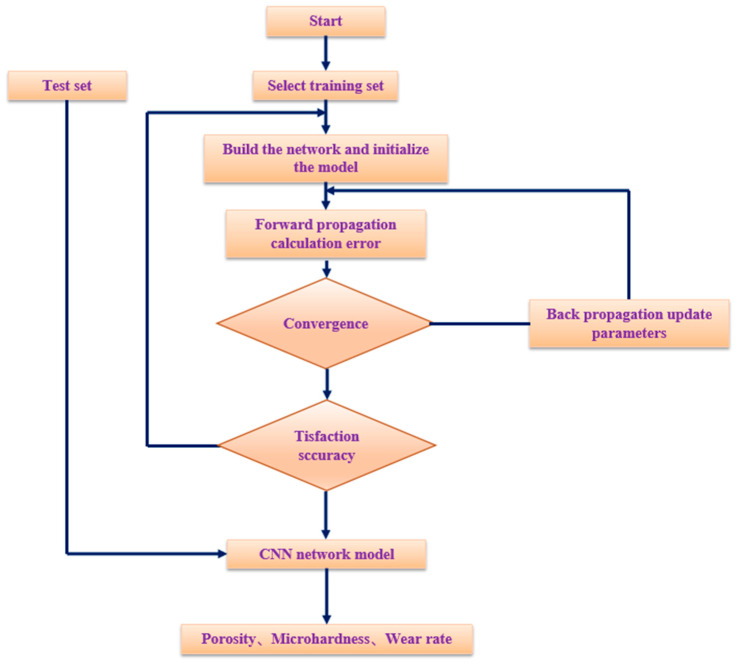
The progress of CNN training.

The CNN model is optimized using Adam’s optimizer, with a learning rate set at 0.00001. The *ReLU* function is selected as the activation function for both convolutional layers, while the categorical cross-entropy function is selected as the loss function. The training process for the CNN is illustrated in Figure 7. To meet the accuracy requirements, the training process is divided into two parts: forward and backward propagation. During forward propagation, the network is constructed and the weights and errors are initialized. Subsequently, the input data are convolved into the fully connected layer and classified. The gradient descent method is applied in backward propagation, and the error is fed back to the convolutional layer based on the loss function through adjusting the network parameters and repeating the aforementioned steps.

## 4. Results and Discussion

### 4.1. Analysis of RF Feature Selection Results

**Figure 8:**
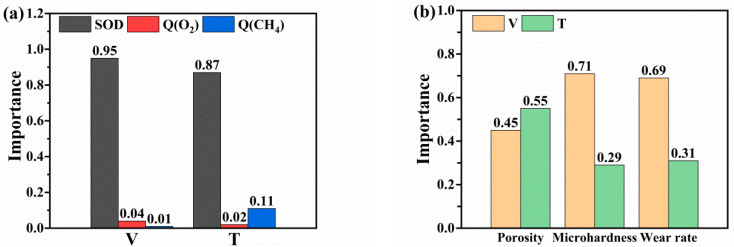
(**a**) The contribution rate of input variables on the velocity and temperature of in-flight particles. (**b**) The contribution rate of input variables on the coating porosity, microhardness, and wear rate.

To select key features for accurate prediction results in further steps, we constructed an RF model herein. This model was used to compute the percentages of feature importance for velocity and temperature in the PINN model and for porosity, microhardness, and wear rate in the CNN model. The RF evaluation determined the contribution of each input variable to each output variable of the PINN and CNN models in further steps. As shown in Figure 8, the feature names were displayed on the horizontal axis, while their corresponding importance was marked on the vertical axis.

Figure 8a illustrates the RF analysis of the PINN model layer, revealing that SOD, Q(O_2_), and Q(CH_4_) are important features affecting the velocity of in-flight particles, while SOD, Q(CH_4_), and Q(O_2_) are features that affect the temperature of these particles. The results demonstrate that SOD exerts the greatest effect on the characteristics of in-flight particles, making 95% and 87% contribution to the velocity and temperature, respectively. However, the effects of Q(O_2_) and Q(CH_4_) on temperature and velocity are limited, which is consistent with the Irregular trend of velocity variation with CH_4_ flow. Figure 8b displays the results of the RF evaluation of the CNN model, indicating that the velocity of in-flight particles exerts a greater effect on the microhardness and wear rate of the coating, making 72% and 69% contributions to these parameters, respectively; the velocity is more important than the temperature characteristics. However, with regard to the coating porosity, the temperature of in-flight particles (55%) is more important than their velocity (45%). It indicated that the porosity of HVOF-sprayed coatings is relatively more sensitive to the melting degree of in-flight particles. Additionally, as shown in Figure 8a, since both the velocity and temperature of in-flight particles are obviously determined by spraying distances, the optimization of spraying distance is an efficient way for controlling the porosities of coatings. Therefore, based on the RF model calculation results, adjusting the velocity range is an effective measure for adjusting the microhardness and wear rate of HVOF-sprayed NiCr–Cr_3_C_2_ coatings. Furthermore, tuning the temperature of impinging particles is more useful to control the porosity of the NiCr–Cr_3_C_2_ coatings.

### 4.2. Analysis of the PINN Training Results

**Figure 9:**
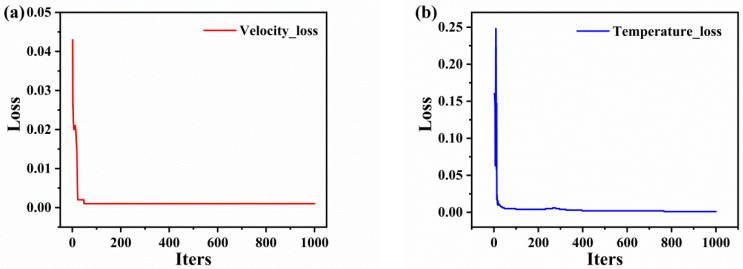
The loss values of PINN training process for predicting velocity (**a**) and temperature (**b**).

**Figure 10:**
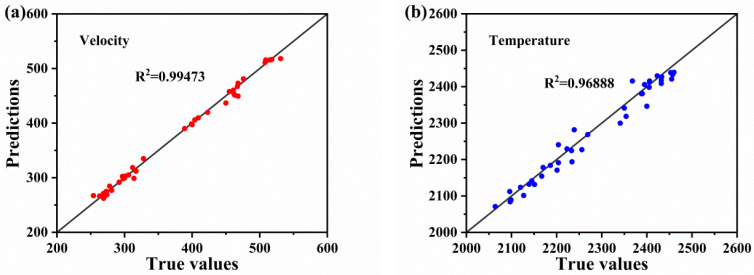
Predicted R^2^ values for velocity (**a**) and temperature (**b**) models.

After training the PINN model, we plotted loss function values for the training set as a function of the epochs (Figure 9). After several rounds of trial and error and parameter adjustments, the training loss values of the PINN network model remained consistently at <0.01 and attained a steady state. Figure 10 displays the fit of the PINN model. Horizontal coordinates, vertical coordinates, and the solid line represent the experimental values, predicted values, and ideal fit results, respectively. The closer the dispersion points are to the solid line, the more accurate is the prediction. As shown in Figure 10a,b, the predicted values of the PINN model training for the temperature and velocity of the in-flight particles are highly similar to the actual values, and the R^2^ values of the model reach 0.99473 and 0.96888, respectively. These results indicate that the PINN model satisfies the training requirements and can be used to predict the velocity and temperature of the in-flight particles.

**Figure 11:**
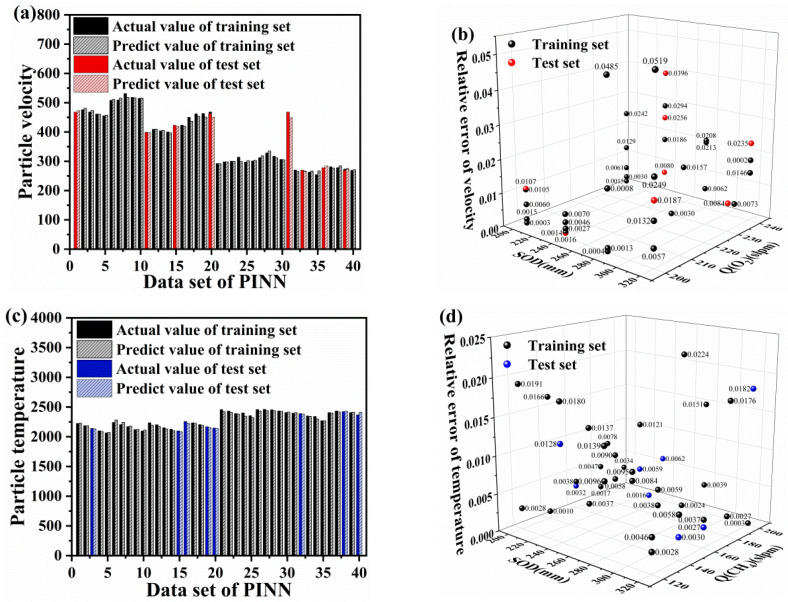
Mean absolute error distribution of the velocity (**a**,**b**) and temperature (**c**,**d**) experimental values and the predicted values from the PINN model.

The mean absolute error (MAE) distribution between the experimental values and the predicted values of the PINN model is shown in Figure 11. It was calculated using Equation (6). Figure 11a,b display the MAE distribution of in-flight particle velocity for the training and validation sets, which were distributed from 0.00033 to 0.05190 with an MAE of 0.01346. Figure 11c,d show the MAE distribution of the in-flight particle temperature, which ranged from 0.00026 to 0.02241 with an MAE of 0.00966. The velocity distribution of in-flight particles was larger and more dispersed than the temperature distribution, indicating that the PINN model was more accurate in predicting temperature owing to the uniform preheating of in-flight particles during the testing process, which led to a more accurate temperature estimation. For in-flight particle velocity values, the model predicted instantaneous velocity during a short period before the in-flight and the MAE was normal (i.e., within the range of acceptability).

To verify the reliability of the PINN model, a randomly selected test set, unrelated to the training and optimization of the PINN model, was used to validate the predicted velocity and temperature performance. Figure 11 shows that the maximum MAEs of the velocity and temperature of in-flight particles in the test set were 0.03962 and 0.01824, respectively, while the minimum MAEs of the velocity and temperature of in-flight particles in the test set were 0.00142 and 0.00026, respectively. In other words, it reveals that the prediction accuracy of temperature and velocity of in-flight particles are 96.89% and 99.47%, respectively. Comparing with in-flight particle predictions based on traditional machine learning models from different thermal spraying routes, including plasma spraying (with 82% and 97% prediction accuracies for mean particle temperature and velocity) [36], cold spraying (with 96.45% predication accuracy for particle velocity) [37], and HVOF (with 99.24% and 99.57% prediction accuracies for mean particle temperature and velocity) [13], the novel hybrid PINN-CNN model exhibited generally more accurate predictions. In addition, results indicated that the predicted and actual values of the model were consistent, which demonstrates that the PINN model was trained and met the prediction requirements for the temperature and velocity of in-flight particles in the HVOF spray process. The model establishes a physical relation to describe the association between HVOF spray process parameters and particle flight characteristics.

### 4.3. Analysis of the CNN Training Results

**Figure 12:**
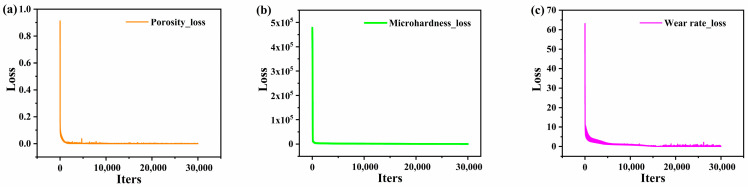
The loss values of CNN training process for predicting porosity (**a**), microhardness (**b**), and wear rate (**c**).

After training the CNN network (Figure 12a–c), we plotted the loss function values of the training set for each training cycle. The results indicated a gradual decrease in the training error with increasing training times; the average training error stabilized at approximately 0.05 after 20,000 training cycles.

**Figure 13:**
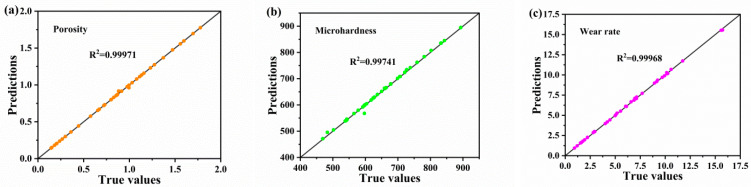
Predicted R^2^ values for predicting porosity (**a**), microhardness (**b**), and wear rate (**c**) models.

**Figure 14:**
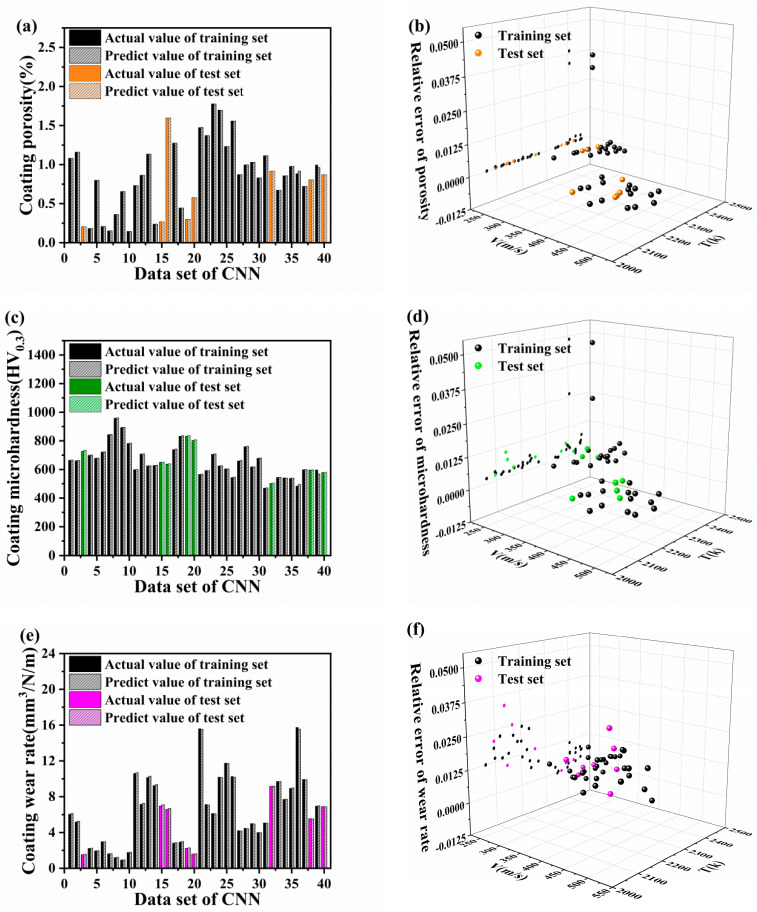
Mean absolute error distribution of the porosity (**a**,**b**), microhardness (**c**,**d**), and wear rate (**e**,**f**) experimental values and the predicted values from the CNN model.

To evaluate the effectiveness of the CNN model, we randomly split the dataset into training and validation sets. The experimental results show that the R^2^ values of the model reached 0.99971, 0.99741, and 0.99968 for the porosity, microhardness, and wear rate, respectively (as shown in Figure 13a–c). These results confirm that the fitting of the model is good. However, the R^2^ values of the CNN model were smaller than those of the PINN model. This difference can be attributed to the ability of the PINN to embed the physical relation equation, which enhances the prediction accuracy and fit of the model. The accuracy of the CNN model was evaluated using MAE (Figure 14a–f). After model training, we reversely normalized the true and predicted values in the training and validation sets. Figure 14 compares the MAEs between experimental values and the predicted values of the CNN model. As shown in Figure 14a,b, the MAEs for the porosity of the coating in the training and validation sets ranged from 0.000002 to 0.03877, with an average of 0.00218. The coating microhardness exhibited maximum and minimum MAEs of 0.04864 and 0.00043, respectively, between true and predicted values, with an average of 0.00513 (Figure 14c,d). Furthermore, the MAEs of the coating wear rate were mostly distributed between 0.000004 and 0.03318, with an average of 0.01078 (Figure 14e,f). The MAE results indicate that the error distribution of the coating wear rate is wider and more dispersed than the distribution of coating microhardness or porosity. This implies that the CNN model accurately predicts the porosity and microhardness of the coating. This is because porosity is a parameter directly obtained after spraying and less influenced by other parameters. Therefore, direct prediction based on particle velocity and temperature provides high accuracy of up to 99%. Furthermore, in case of coating microhardness, the error bars are larger, the CNN data exhibit fluctuations, and the test data are affected by specimen size, the degree of particle flattening, and residual stress. The accuracy of data predicted directly on the basis of particle velocity and temperature is lower than that predicted on the basis of porosity. The coating wear rate is influenced by the porosity, microhardness, and roughness of the sample and exhibits a substantial relation with the surface condition of the specimen post treatment. Therefore, it is understandably influenced by the crossover of parameters, and the prediction accuracy of the model is low.

Figure 14 demonstrates the reliability of the CNN model through evaluating its prediction performance on the test set. The porosity, microhardness, and wear rate have maximum MAEs of 0.00063, 0.01009, and 0.03318, respectively. The minimum MAEs for porosity, microhardness, and wear rate are 0.00029, 0.00146, and 0.00052, respectively. These results indicated that the predication accuracy for coatings’ porosity, microhardness and wear rate are 99.97%, 99.74%, and 99.97%, respectively. As a comparison, based on the Generative Adversarial Network (GAN) model, the prediction accuracy for coating characteristics via suspension plasma spraying is only 80% [24]. Consequently, the hybrid PINN-CNN model also possess advantages with excellent prediction accuracy for coating performances.

## 5. Conclusions

Herein, a new hierarchical neural network model based on the PINN and CNN has been proposed to simulate the relation between HVOF spray process parameters, in-flight particle characteristics, and NiCr–Cr_3_C_2_ coating properties. It should be mentioned that the development of the novel hybrid PINN-CNN model could be beneficial for reducing the overfitting problem from the conventional ANN model, as well as compensating for the poor transferability of traditional machine learning techniques due to the usage of PINN model. The main contributions of the proposed approach can be summarized as follows: First, an RF model was developed for feature selection to assess the importance of each feature. Results demonstrate that SOD exerts the greatest effect on the velocity and temperature of in-flight particles and that the velocity of in-flight particles affects the microhardness and wear rate of the coating; the temperature of in-flight particles is more important for the porosity of the coating than their velocity. Second, this study proposes a novel approach: the prediction of HVOF-sprayed coatings based on the hybrid PINN-CNN model. The PINN model can predict the velocity and temperature of in-flight particles via training and optimization, with MAEs of 0.01346 and 0.00966, respectively. Third, the CNN model considers the velocity and temperature of in-flight particles predicted by the PINN as the input and predicts the porosity, microhardness, and wear rate. The MAEs of the CNN model are 0.00218, 0.00513, and 0.01078 for the porosity, microhardness, and wear rate, respectively, which achieve good prediction accuracy and less overfitting. The hybrid PINN-CINN model prediction errors are 1% overall, and the R^2^ is >96%, which possessed comparable or even better prediction accuracies for both in-flight particle characteristics and coating performances compared to traditional ML models. Moreover, the development of the novel hybrid PINN-CNN model is beneficial for reducing the overfitting problem from the conventional ANN model, as well as compensating for the poor transferability of traditional machine learning techniques due to the usage of the PINN model. All in all, the hierarchical neural network model is not only capable of effectively realizing the prediction of in-flight particle behavior and coating performances as well as its influencing factors, but also providing a theoretical basis for preparing and optimizing high-performance HVOF-sprayed coatings.

## Figures and Tables

**Table 1:** Spraying parameters for NiCr-Cr_3_C_2_ coatings in the HVOF process.

Parameters	Scope
O_2_ flow (slpm)	200–240
CH_4_ flow (slpm)	120–200
Air flow (slpm)	300
Carrier gas flow (slpm)	40
stand-off distance (mm)	200–320
Spray gun speed (mm/s)	400
Powder feeding speed (g/min)	30

**Table 2:** Spraying parameters, characteristics of in-flight particles, and coating performances.

No.	Q(CH_4_) (slpm)	SOD (mm)	Q(O_2_) (slpm)	V (m/s)	T (K)	MH (HV_0.3_)	WR × 10^−5^ (mm^3^/N/m)	PO (% Area)
1	120	200	200	467	2223	664	6.039	1.08
2	120	200	240	508	2239	721	2.962	0.206
3	120	240	200	389	2234	598	10.587	0.731
4	120	240	240	423	2256	637	6.585	1.597
5	120	280	200	292	2455	565	15.589	1.473
6	120	280	240	301	2457	543	10.249	1.558
7	120	320	200	269	2395	469	5.061	1.113
8	120	320	240	274	2405	483	15.732	0.883
9	140	200	200	476	2186	661	5.175	1.159
10	140	200	240	509	2204	843	1.624	0.151
11	140	240	200	409	2201	707	7.142	0.863
12	140	240	240	450	2233	738	2.802	1.274
13	140	280	200	298	2432	592	7.122	1.372
14	140	280	240	312	2460	659	4.203	0.871
15	140	320	200	270	2388	502	9.154	0.915
16	140	320	240	281	2432	598	9.927	0.721
17	160	200	200	468	2139	727	1.516	0.204
18	160	200	240	531	2170	958	1.197	0.361
19	160	240	200	404	2151	625	10.144	1.134
20	160	240	240	462	2204	832	2.926	0.444
21	160	280	200	300	2390	706	6.122	1.777
22	160	280	240	328	2453	759	4.467	0.996
23	160	320	200	265	2350	544	9.698	0.671
24	160	320	240	278	2423	596	5.54	0.803
25	180	200	200	461	2099	699	2.217	0.182
26	180	200	240	518	2120	893	0.926	0.655
27	180	240	200	400	2127	628	9.235	0.237
28	180	240	240	463	2167	833	2.221	0.299
29	180	280	200	314	2400	624	10.172	1.697
30	180	280	230	317	2433	618	4.997	1.03
31	180	320	200	263	2341	541	7.693	0.856
32	180	320	233	273	2406	597	6.941	0.995
33	186	200	240	515	2096	781	1.764	0.144
34	188	240	240	468	2145	802	1.59	0.576
35	200	200	200	455	2064	678	1.944	0.799
36	200	240	200	399	2097	650	6.955	0.266
37	200	280	200	297	2354	604	11.741	1.231
38	200	280	225	306	2406	678	4.001	0.83
39	200	320	200	254	2269	538	8.92	0.977
40	200	320	230	269	2368	578	6.881	0.87

## Data Availability

Not applicable.
